# Role of echocardiography in diagnosis and risk stratification in heart failure with left ventricular systolic dysfunction

**DOI:** 10.1186/1476-7120-5-34

**Published:** 2007-10-02

**Authors:** Quirino Ciampi, Bruno Villari

**Affiliations:** 1 Division of Cardiology, Fatebenefratelli Hospital, Benevento, Italy

## Abstract

Heart failure (HF) is a complex clinical syndrome that can result from any structural or functional cardiac disorder that impairs the ability of the ventricle to fill with or eject blood. Echocardiography represents the "gold standard" in the assessment of LV systolic dysfunction and in the recognition of systolic heart failure, since dilatation of the LV results in alteration of intracardiac geometry and hemodynamics leading to increased morbidity and mortality.

The functional mitral regurgitation is a consequence of adverse LV remodelling that occurs with a structurally normal valve and it is a marker of adverse prognosis.

Diastolic dysfunction plays a major role in signs and symptoms of HF and in the risk stratification, and provides prognostic information independently in HF patients and impaired systolic function.

Ultrasound lung comets are a simple echographic sign of extravascular lung water, more frequently associated with left ventricular diastolic and/or systolic dysfunction, which can integrate the clinical and pathophysiological information provided by conventional echocardiography and provide a useful information for prognostic stratification of HF patients.

Contractile reserve is defined as the difference between values of an index of left ventricular contractility during peak stress and its baseline values and the presence of myocardial viability predicts a favorable outcome. A non-invasive echocardiographic method for the evaluation of force-frequency relationship has been proposed to assess the changes in contractility during stress echo.

In conclusion, in HF patients, the evaluation of systolic, diastolic function and myocardial contractile reserve plays a fundamental role in the risk stratification. The highest risk is present in HF patients with a heart that is weak, big, noisy, stiff and wet.

## Background

Heart failure (HF) is a complex clinical syndrome that can result from any structural or functional cardiac disorder that impairs the ability of the ventricle to fill with or eject blood [1].

The syndrome of HF is a common manifestation of the later stages of various cardiovascular diseases, including coronary artery disease, hypertension, valvular disease, and primary myocardial disease.

The cardinal manifestations of HF are dyspnea and fatigue, which may limit exercise tolerance, and fluid retention, which may lead to pulmonary congestion and peripheral edema. Both abnormalities can impair the functional capacity and quality of life of affected individuals, but they do not necessarily dominate the clinical picture at the same time. Some patients have exercise intolerance but little evidence of fluid retention, whereas others complain primarily of edema and report few symptoms of dyspnea or fatigue [2,3].

Approximately 50% of HF patients present with evidence of left ventricular systolic dysfunction manifested as a low left ventricular ejection fraction [4].

HF is considered a progressive disorder that can be represented as a clinical continuum. The American College of Cardiology/American Heart Association (ACC/AHA) updated 2005 guidelines for the management of chronic HF identify 4 stages in this continuum. Stage A: risk for HF but without structural heart disease or symptoms of HF; Stage B: structural heart disease but without signs or symptoms of HF; Stage C: structural heart disease with prior or current symptoms of HF; Stage D: refractory HF [5]. The number of patients with LV systolic dysfunction in stage B is estimated to be 4 times greater than in stages C and D combined [6]. These patients remain at risk for significant morbidity and mortality and the subsequent development of symptomatic HF. Because substantial evidence indicates that pharmacological intervention may have an effect on the risk of progression to HF and death, identification of patients who are asymptomatic would then appear to be a priority.

**Figure 1:**
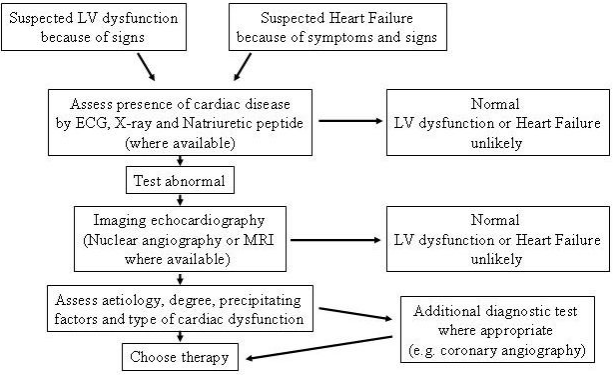
Algorithm of diagnosis of heart failure or left ventricular dysfunction.

ACC/AHA guidelines [5] as well as ESC guidelines [7] state that echocardiography is the single most useful test in the diagnosis of heart failure since structural abnormality, systolic dysfunction, diastolic dysfunction, or a combination of these abnormalities needs to be documented in patients who present with resting or/and exertional symptoms of heart failure to establish a definitive diagnosis of heart failure (Figure 1). It is important to demonstrate an objective evidence of structural or functional abnormalities to explain patient's symptoms of heart failure since symptoms of heart failure are not specific and more than a third of patients with a clinical diagnosis of heart failure may not actually have heart failure [8].

European Society of Cardiology guidelines [5] recommend chest x-ray for the evaluation of patients with suspected LV dysfunction because of signs or symptoms. In particular, chest x-ray is useful to detect the presence of cardiac enlargement and pulmonary congestion. However, echocardiography represents the "gold standard" in the assessment of LV systolic dysfunction, it can certainly do better than chest x-ray for cardiac enlargement, and may also provide direct imaging of pulmonary congestion. In addition, it is important to consider the disadvantage of radiation exposure in situations, such as heart failure, when serial assessment is mandatory [9,10]. Current protection standard and practices are based on the premise that any ionising radiation dose, no matter how small, can result in detrimental health effects [11]. These include long-term development of cancer and genetic damage [12]. For the purposes of radiation protection, the dose-response curve for radiation-induced cancer is assumed to be linear at low doses, with no minimum threshold [13].

Owing to high cost and the need for skilled technicians, in the absence of some other clinical indication, such as a prior myocardial infarction, abnormality of electrocardiogram, family history of cardiomyopathy, or HF symptoms, routine comprehensive echocardiography cannot be recommended currently.

### Left ventricular systolic dysfunction (the *weak *heart)

Heart failure due to systolic dysfunction is relatively easy to diagnose by echocardiography which demonstrates a dilated left ventricle with a reduced ejection fraction. In systolic heart failure, however, echocardiography has many other roles beyond the recognition of systolic heart failure since dilatation of the LV results in alteration of intracardiac geometry and hemodynamics leading to increased morbidity and mortality [14].

**Figure 2:**
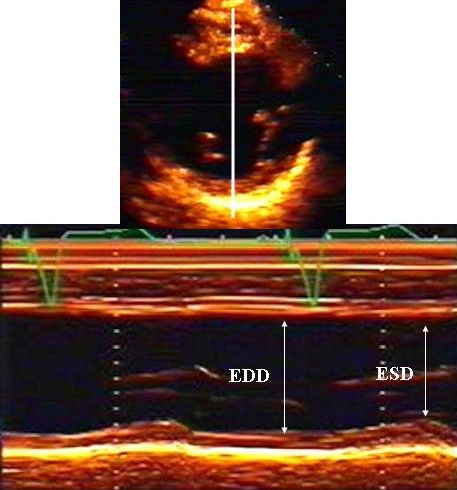
Measurement of left ventricular end-diastolic diameter (EDD) and end-systolic diameter (ESD) from M-mode (down), guided by a parasternal short axis image (upper) to optimize medial-lateral beam orientation.

Left ventricular dimensions, volumes and wall thicknesses are echocardiographic measurements widely used in clinical practice and research. To obtain accurate linear measurements of interventricular septal and posterior wall thicknesses and LV internal dimension, recordings should be made from the parasternal long-axis acoustic window [15]. It is recommended that LV internal diameters and wall thicknesses be measured at the level of the LV minor axis, approximately at the mitral valve leaflet tips (Figure 2). However, it should be recognized that even with 2-dimensional echo (2D) guidance, it may not be possible to align the M-mode cursor perpendicular to the long axis of the ventricle which is mandatory to obtain a true minor axis dimension measurement. Alternatively, chamber dimension and wall thicknesses can be acquired from the parasternal short-axis view using direct 2D measurements or targeted M-mode echocardiography provided that the M-mode cursor can be positioned perpendicular to the septum and LV posterior wall [15].

**Figure 3:**
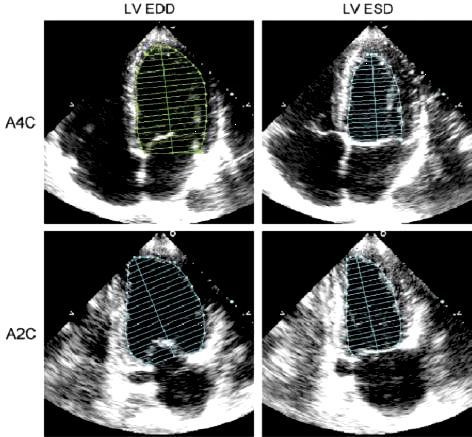
2-D measurements for volume calculations using the biplane method of discs (modified Simpson's rule), in the apical four-chamber (A4C) and apical two-chamber (A2C) views at end diastole (LV EDD) and at end-systole (LVESD). The papillary muscles should be excluded from the cavity in the tracing.

In order to obtain volumetric measurements the most important views for 2D quantization are the mid-papillary short-axis view and the apical four and two-chamber views [15]. Volumetric measurements require manual tracing of the endocardial border. The papillary muscles should be excluded from the cavity in the tracing. Accurate measurements require optimal visualization of the endocardial border in order to minimize the need for extrapolation. The most commonly used 2-D measurement for volume measurements is the biplane method of discs (modified Simpson's rule) [16]. The principle underlying this method is that the total LV volume is calculated from the summation of a stack of elliptical discs. The height of each disc is calculated as a fraction (usually one-twentieth) of the LV long axis based on the longer of the two lengths from the two and four-chamber views (Figure 3). End-diastole can be defined at the onset of the QRS, but is preferably defined as the frame following mitral valve closure or the frame in the cardiac cycle in which the cardiac dimension is largest. End-systole is best defined as the frame preceding mitral valve opening or the time in the cardiac cycle in which the cardiac dimension is smallest in a normal heart. LV size and performance are still frequently visually estimated. However, qualitative assessment of LV size and function may have significant inter-observer variability and is a function of interpreter skill. Therefore, it should regularly be compared to quantitative measurements, especially when different views qualitatively suggest different degrees of LV dysfunction. Similarly, it is also important to cross-check quantitative data using the "eye-ball" method, to avoid overemphasis on process-related measurements, which at times may depend on structures seen in a single still-frame. While these inaccuracies in the measurement of LV volume and ejection fraction have been considered inevitable and of minor clinical importance in the past, in most situations accurate measurements are required, particularly when following the course of a disease with serial examinations [17].

Several three dimensional (3D) echocardiographic techniques became available to measure LV volumes and ejection fraction. Regardless of which acquisition or analysis method is used, 3D echocardiography does not rely on geometric assumptions for volume/mass calculations and is not subject to plane positioning errors [18,19]. Studies comparing 3D echocardiographic LV volumes to other gold-standards such as magnetic resonance imaging, have confirmed 3D echocardiography to be accurate. Compared to magnetic resonance data, LV volumes calculated from 3D echocardiography showed significantly better agreement (smaller bias), lower scatter and lower intra- and inter-observer variability than 2-D echocardiography [20]. Current limitations include the requirement of regular rhythm, relative inferior image quality of real-time 3D echocardiography compared to 2D images, and the time necessary for off-line data analysis.

The LV systolic function is normal if ejection fraction is >55%, and considered severely abnormal if ejection fraction is <30% (Table 1).

**Table 1:** The risk factor in patients with systolic heart failure

	Abnormality		
	Mild	Moderate	Severe

LV end-systolic volume (ml/m^2^)	<30	30–60	>60
LV ejection fraction (%)	45–54	44–30	<30
Transmitral LV diastolic dysfunction (E/A)	Grade I	Grade II-III	Grade IV
Tissue Doppler diastolic dysfunction (E/E')	<8	8–15	>15
Ultrasound Lung Comets	5–15	16–30	>30
Mitral regurgitation (Jet/left atrial)	<20	20–40	>40

For the analysis of the regional function, in 2002 the American Heart Association Writing Group on Myocardial Segmentation and Registration for Cardiac Imaging [21], in an attempt to establish segmentation standards applicable to all types of imaging, recommended a 17-segment model. This model consists of six segments at both basal and mid-ventricular levels and four segments at the apex. The 17th segment, the "apical cap" is the segment beyond the end of the LV cavity.

### Left ventricular remodeling (the *big *heart)

Transition to pathologic remodeling is heralded by progressive ventricular dilatation, distortion of cavity shape and disruption of the normal geometry of the mitral annulus and subvalvular apparatus resulting in mitral regurgitation [22].

Pathologic LV remodeling is the final common pathway to heart failure, whether the initial stimulus is chronic pressure or chronic volume overload, genetically determined cardiomyopathy or myocardial infarction. Changes in LV size and geometry due to pressure overload reflect the dominant underlying hemodynamic alterations associated with blood pressure elevation. The pressure-overload pattern of concentric hypertrophy is uncommon in otherwise healthy hypertensive individuals and is associated with high systolic blood pressure and high peripheral resistance. In contrast, the volume-overload pattern is characterized by eccentric LV hypertrophy and it is associated with normal peripheral resistance but high cardiac index consistent with excess circulating blood volume [23,24].

While LV remodeling in patients with chronic systemic hypertension, chronic valvular regurgitation and primary cardiomyopathies has been described, the transition to heart failure is less well known because the time course is so prolonged. By contrast, the time course from myocardial infarction to heart failure is shorter and has been clearly documented. A unique form of remodeling occurs following myocardial infarction due to the abrupt loss of contracting myocytes. Early expansion of the infarct zone is associated with early LV dilatation as the increased regional wall stress is redistributed to preserve stroke volume. The extent of early and late post-infarction remodeling is determined by a number of factors, including size and location of infarction, activation of the sympathetic nervous system, and up-regulation of the renin/angiotensin/aldosterone system and natriuretic peptides [25].

The traditional quantitative echocardiographic measurements recommended to evaluate LV remodeling included estimates of LV volumes either from biplane images as advocated by the American Society of Echocardiography [11]. LV volumes and derived ejection fraction have been demonstrated to predict adverse cardiovascular events at follow-up, including death, recurrent infarction, heart failure, ventricular arrhythmias and mitral regurgitation in numerous post-infarction and heart failure trials [25,26].

### Mitral regurgitation (the *noisy *heart)

Mitral regurgitation is a common finding in HF patients. In patients with dilated and ischemic cardiomyopathy, the mitral regurgitation is typically functional and reflects geometric distortions of LV chamber, which displaces the normal valve and subvalvar closing mechanisms [27]. This functional mitral regurgitation is a consequence of adverse LV remodelling and increased sphericity of the chamber. Functional mitral regurgitation is typically dynamic, occurs with a structurally normal valve and it is a marker of adverse prognosis [28,29]. Mitral regurgitation further increases the propensity for severity of HF. In fact, LV dilatation begets mitral regurgitation and mitral regurgitation begets further LV dilatation, progressive remodeling and contractile dysfunction [30].

The presence and degree of mitral regurgitation complicating HF are unrelated to the severity of systolic dysfunction. Local LV remodeling (apical and posterior displacement of papillary muscles) leads to excess valvular tenting independent of global LV remodeling. In turn, excess tenting and loss of systolic annular contraction are associated with larger mitral regurgitation. Tenting is characterized by insufficient systolic leaflet body displacement toward the annulus, with coaptation limited to leaflet tips. Valvular tenting area was measured by the area enclosed between the annular plane and mitral leaflets from the parasternal long-axis view at early and late systole. The distance between leaflet coaptation and the mitral annulus plane at early and end systole measured displacement of mitral coaptation toward the LV apex [27,30,31].

In a study with 3-D echocardiography [32], the authors demonstrated that in ischemic cardiomyopathy–mitral regurgitation, the LV chamber and mitral annulus were less enlarged than in dilated cardiomyopathy despite the presence of virtually the same grade of mitral regurgitation. In ischemic cardiomyopathy–mitral regurgitation, the pattern of mitral valve deformation from the medial to the lateral side of the mitral valve was asymmetrical (significant tethering of both leaflets on the medial side, but significant tethering of only the posterior leaflet on the lateral side), whereas it was symmetrical in dilated cardiomyopathy – mitral regurgitation (significant tethering of both leaflets on both sides). Hence, 3-D echocardiography is a helpful tool for differentiating the geometry of the mitral apparatus between these 2 different types of functional mitral regurgitation. This finding suggests that in ischemic cardiomyopathy–mitral regurgitation, mitral regurgitation severity is mainly associated with regional geometry of the LV chamber rather than global geometry.

Stress echocardiography in the form of exercise or pharmacologic protocols can be useful in the assessment of mitral regurgitation, and it can play several roles in the assessment of the behaviour of mitral valve in HF patients [33]. In symptomatic patients with LV dysfunction and a clinical picture suspicious for new or worsening mitral regurgitation, but not evident at resting echo examination, exercise echocardiography can demonstrate a worsening of mitral regurgitation which helps to correlate this scenario with the patient's symptoms [34]. LV contractility, in presence of mitral regurgitation, can impair or improve during exercise with consequent modification of mitral regurgitation. The presence of myocardial contractile reserve is related to decrease of mitral regurgitation [35], whereas generally a fall in stroke volume is associated with an increase in mitral regurgitant volume is associated with an increase in mitral regurgitant volume during isometric exercise, which increases systemic resistances and thereby afterload [36]. These observations support the concept of presence of a vicious circle between LV function and behaviour of mitral regurgitation. Therefore, to study these patients with exercise echocardiography may be important for assessing the response of mitral regurgitation to medical therapy and for the following prognostic implications. Indeed, in patients with ischemic mitral regurgitation and LV dysfunction, quantitative assessment of exercise-induced changes in the degree of mitral regurgitation provides independent prognostic information [37].

### Left ventricular diastolic dysfunction (the *stiff *heart)

Diastolic dysfunction refers to the presence of abnormalities in filling of the ventricle [38].

LV filling consists series of hemodynamic events that are affected by multiple intrinsic and extrinsic factors. The initial diastolic event is myocardial relaxation, an active energy-dependent process, that causes a decrease rapidly in the pressure of left ventricle after the end of contraction and during early diastole. Doppler echocardiography is an extremely sensitive tool for the detection and measurement of pressure gradient (driving force) from the left atrium to the LV during diastole [39-41].

When LV pressure falls below left atrial pressure, the mitral valve opens and rapid early diastolic filling begins. Approximately 80% of LV filling normally occurs during this phase. As a result of rapid filling, LV pressure increases and exceeds left atrial pressure, and this loss of positive driving force results in deceleration of mitral flow velocity. A positive transmitral pressure gradient and flow are again created by atrial contraction during late diastolic.

Mitral flow velocities are obtained by pulsed-wave Doppler echocardiography with the sample volume located between the tips of mitral leaflets during diastole. Initial classification of diastolic filling is made from peak velocity of early rapid filling wave (E), peak velocity of late filling wave caused by atrial contraction (A), and E/A ratio. Diastolic filling pattern is characterized further by measuring deceleration time, which is the interval from the peak of E velocity to its extrapolation to the baseline [39-41].

In the early stages of diastolic dysfunction, impaired (delayed) relaxation of the left ventricle predominates, and this decreases early diastolic filling. An abnormal relaxation pattern is seen on the mitral flow velocity curve and consists of a low E velocity, prolongation of the deceleration time and increased filling at atrial contraction. The deceleration time is characteristically prolonged because it takes longer for left atrial and LV pressures to be equilibrated with a slower and continued fall in LV pressure until mid to late diastole and a reduced rate of filling during early diastole (E). At this stage, there is little if any increase in rest left ventricular diastolic filling pressure [42].

With disease progression, left atrial pressure increases, thus increasing the driving pressure across the mitral valve. There is a gradual increase in the E velocity on the mitral flow velocity curve. As effective operative compliance decreases, the deceleration time shortens, and a pseudonormal pattern appears. In more advanced disease, the left atrial pressure is higher and ventricular compliance is poor, producing a restriction to filling pattern.

On the basis of this progression of disease patterns, we would like to propose a grading system for the severity of diastolic dysfunction as assessed with Doppler echocardiography [43]. Using a scale of I to IV, grade I identifies a patient with an abnormal relaxation pattern and minimal or no symptoms of heart failure at rest. Patients with grade I diastolic dysfunction may develop dyspnea with moderate to extreme exertion or may develop symptoms of heart failure if the contribution from atrial contraction is lost, as occurs with development of atrial fibrillation. With grade II diastolic dysfunction, there is a pseudo-normalization pattern on the mitral flow velocity curves and increased filling pressures at rest, producing symptoms with mild to moderate exertion. Patients with grade III diastolic dysfunction have a restrictive reversible filling pattern on the mitral flow velocity curves, severe increase in filling pressures and symptoms at rest or with minimal exertion. Some patients with severe abnormalities of ventricular compliance and end-stage heart disease maintain a severe restrictive pattern even after aggressive diuresis. Grade IV is characterized by a restrictive irreversible filling pattern. Previous works showed that restrictive LV filling pattern, in HF patients, is associated with a more severe clinical and hemodynamic status and with increased mortality: it is an independent prognostic marker [44-47].

Tissue Doppler imaging (TDI) is an echocardiographic technique with the capacity to quantify systolic and diastolic functions both globally and regionally [48,49]. TDI is useful for the detection of left ventricular systolic and diastolic dysfunction, because it integrates detailed information of regional function to estimate global cardiac function. Systolic function is in fact one of the most important determinants of diastolic function: in fact systolic and diastolic functions are closely coupled in the cardiac cycle and both are energy-dependent processes. Yu et al. [50] demonstrated that, in patients with diastolic heart failure, there is objective evidence of impaired left ventricular systolic function as demonstrated by TDI. In these patients, the regional function, assessed by mitral anulus peak systolic velocity, was decreased. The mitral anulus peak systolic velocity appears to be a more sensitive index of early systolic dysfunction than ejection fraction and, hence, in a proportion of these patients the systolic function was labeled as "normal" by conventional methods. This indicates the common coexistence of systolic and diastolic dysfunction in a spectrum of different severity in the pathophysiological process of heart failure.

The velocity of annular motion reflects shortening and lengthening of the myocardial fibers along a longitudinal plane. The mitral annulus early diastolic velocity (Ea) is an index of LV relaxation that may not be influenced by left atrial pressure. Ea is lower at the septal annulus (normal >10 cm/sec) compared to the lateral annulus (normal >15 cm/sec). Using early diastolic velocity of mitral anulus, Nagueh et al [51] identified patients with relaxation abnormalities independent of the filling pressures and, consequently, differentiated the pseudonormal from the normal LV filling pattern. Furthermore, the ratio of the transmitral E velocity of mitral flow and early diastolic velocity of mitral anulus is related significantly with pulmonary capillary Wedge pressure, suggesting that this measurement can be used as an index of filling pressures.

### Ultrasound lung comets (the *wet *heart)

The interstitial pulmonary edema is a key parameter in the management of patients with chronic heart failure and an early warning sign of impending acute heart failure. The objective diagnosis is traditionally based on chest radiographic findings which, when performed at the bedside, may be difficult to interpret, and may have weak correlations with extravascular lung water [52,53]. The lung is considered poorly accessible using ultrasound since air prevents the progression of the ultrasound beam with production of reverberation artifacts under the lung surface [54]. The "comet-tail image" is an echographic image detectable at bedside with ultrasound probes positioned over the chest [55]. This image consists of multiple comet tails fanning out from the lung surface originating from water-thickened interlobular septa. Functionally, they are a sign of distress of the alveolar-capillary membrane, often associated with reduced ejection fraction and increased pulmonary wedge pressure and they probably represent an ultrasonic equivalent of radiologic Kerley B lines [56]. These features make ultrasound lung comets an appealing simple clinically useful sign for detecting and quantifying extravascular lung water [57] in patients with known or suspected heart failure. Thus, their presence and number permit quantification of the excess of extravascular lung water, providing an indirect measurement of wedge pressure. Moreover, it is sufficiently sensitive and accurate to detect pulmonary interstitial edema even before it becomes apparent clinically. This turns into an advantage because these images are detectable at a very early stage of pulmonary edema, appearing below the threshold of alveolar edema [58]. In fact, alveolar edema is always preceded by interstitial edema, a constant feature of pulmonary edema, the radiologic diagnosis of which is difficult at bedside.

Bedside chest ultrasound has numerous clinical advantages. Recognition of the comet-tail image provides immediate noninvasive information; it can be performed at bedside also with an unsophisticated hand-held device; it is very simple to interpret and easy to quantify; it is not dependent on cardiac acoustic window or patient decubitus; the learning curve is short; and due to the no-ionizing nature of the examination, it is useful in following up the patient over time and tailoring therapy. Ultrasound lung comets are easy both to obtain and to measure (learning curve of <10 examinations, 30 minutes) and fast to perform (<3 minutes), require very limited technology, even without a second harmonic or Doppler and are not restricted by cardiac acoustic window limitations or patient decubitus [56,57].

In patients with acute heart failure were directly related to NYHA functional class, BNP levels and severity of diastolic dysfunction and inversely related to ejection fraction [59]. The same group demonstrated that the 16-months event-free survival was highest in patients without ultrasound lung comets and lowest in patients with severe (>30) ultrasound lung comets at entry. At univariate analysis, were more powerful predictors than other echocardiographic variables [60].

Finally, ultrasound lung comets are a simple echographic sign of extravascular lung water, more frequently associated with left ventricular diastolic and/or systolic dysfunction. Ultrasound lung comets can usefully integrate the clinical and pathophysiological information provided by conventional 2D and Doppler echocardiography in patients with known or suspected heart failure and dyspnea as a presenting symptom and provide a useful information for prognostic stratification of patients with dyspnea.

### Stress echo: evaluation of myocardial viability

Despite the wealth evidence that favor use of stress echocardiography in patients with dilated cardiomyopathy [61-65], there is no clear algorithm about its use in risk stratification and therapeutic strategy. The reasons for this are not clear, but probably reflect the lack of standardized protocol and measurements of left ventricular contractile reserve. Unlike protocols for stress-echocardiography for coronary artery disease, there is no consensus about the protocol to be used in patients with left ventricular systolic dysfunction. The majority of authors have used either low- or high dose dobutamine echocardiography.

All studies on stress-echocardiography in HF patients measured contractile reserve of the left ventricle. Contractile reserve is defined as the difference between values of an index of left ventricular contractility during peak stress and its baseline values. There is no consensus on what index to use. Ejection fraction is the most frequently used index of left ventricular performance. However, it may not accurately reflect left ventricular contractility since it is heavily dependent on loading conditions which is particularly important in patients with heart failure for the following reasons [66]. First of all, mitral regurgitation is frequent in these patients, and can lead to overestimation of left ventricular contractility due to rise in ejection fraction caused by changes in loading conditions (higher preload, lower afterload) [67]. Secondly, activation of neuroendocrine compensatory mechanisms may increase afterload, which in turn may subsequently decrease ejection fraction [68]. Thirdly, left ventricular preload is dependant upon interventricular interaction which is exaggerated in cases of pulmonary hypertension [69] a frequent finding in HF patients. It is generally accepted that increase in ejection fraction by ≥ 5% or change from baseline ejection fraction by ≥ 20% during stress-echocardiography identifies patients with preserved left ventricular contractile reserve and better prognosis. Ejection fraction should be assessed by Simpson biplane formula.

Wall motion score index assessed in a standard manner [16] was frequently used to assess the presence of myocardial viability in dilated cardiomyopathy [62,70]. The major potential drawback for use of this index is semiquantitive assessment of wall motion, which is even more subjected to inter- and intraobserver variability in heart failure patients due to pre-existing wall motion abnormalities and substantial number of patients with left bundle branch. It has been suggested that dobutamine induced change in wall motion score index of ≥ 0.44 identifies patients who will do better during the follow-up.

The assessment of force-frequency relationship is a theoretically robust approach for evaluating left ventricular contractility, which has been deployed clinically using invasive, complex and technically demanding methods [71,72]. Recently, a non-invasive echocardiographic method has been proposed to assess the changes in contractility during exercise echo [73-75]. To build the force-frequency relationship, the force is determined at each step as the ratio of the systolic pressure (cuff sphygmomanometer)/end-systolic volume index (biplane Simpson rule/body surface area). This novel method is based upon the proven assumption that positive inotropic interventions are mirrored by smaller end-systolic volumes and higher end-systolic pressures. Abnormal responses were identified on the basis of the lower absolute value of force-frequency relationship slope and of the lower critical heart rate in the presence of an abnormal biphasic response of force-frequency relationship over increasing frequencies. The force-frequency relationship is defined up-sloping (normal) when peak exercise systolic pressure/end-systolic volume index is higher than baseline and intermediate stress values. The abnormal responses are: biphasic, with an initial up-sloping followed by a later down-sloping trend, when peak exercise systolic pressure/end-systolic volume index is lower than intermediate stress values; flat or negative, when peak exercise systolic pressure/end-systolic volume index is equal to or lower than baseline stress values [75]. The critical heart rate is defined as the heart rate at which systolic pressure/end-systolic volume index reaches the maximum value during progressive increase in heart rate; in biphasic pattern, the critical heart rate is the heart rate beyond which systolic pressure/end-systolic volume index has declined by 5%; in a negative pattern the critical heart rate is the starting heart rate [76].

**Figure 4:**
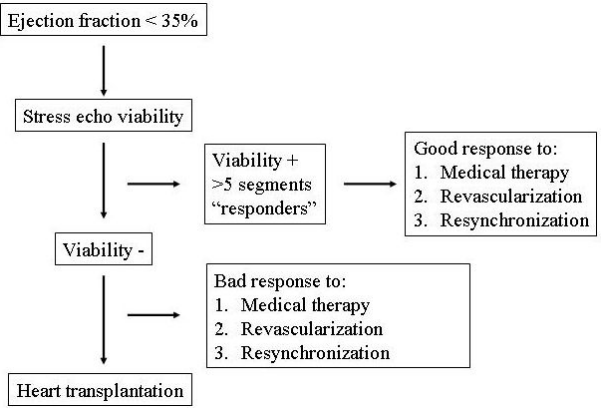
Role of stress echo viability in heart failure.

In HF patients and reduced ejection fraction, the presence of viability play an important role in the response to therapy and is a key determinant in the prognosis not only in patients with stunned myocardium after acute myocardial infarction [77], but also in patients with dilated cardiomyopathy [62,78,79]. In fact the presence of myocardial viability predicts a good response in ischemic HF cardiomyopathy that underwent to myocardial revascularization therapy [80], and in HF patients and LV dyssynchrony (left bundle branch block and QRS >120 ms) that underwent resynchronization therapy using biventricular pacing and in HF patients that started or increased medical therapy [81]. The absence of myocardial viability is related to a bad response to revascularization therapy in ischemic cardiomyopathy, to resynchronization therapy and to medical therapy. The unavoidable way for HF patients with reduced systolic function without myocardial viability during stress echo is the heart transplantation (Figure 4).

### Evaluation of the risk in systolic heart failure

**Figure 5:**
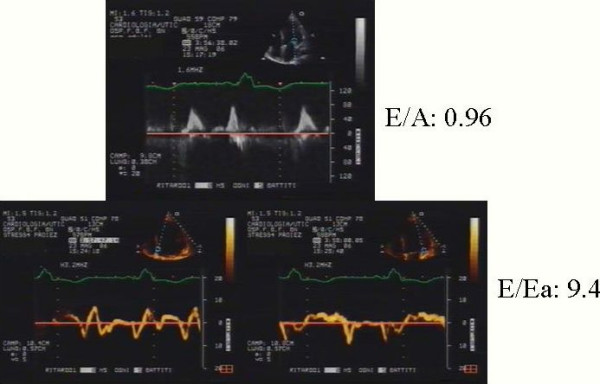
Transmitral LV filling pattern (up); mitral annulus pulsed Tissue Doppler velocity at septum (left panel) septal (down left) and lateral wall (down right) corner.

**Figure 6:**
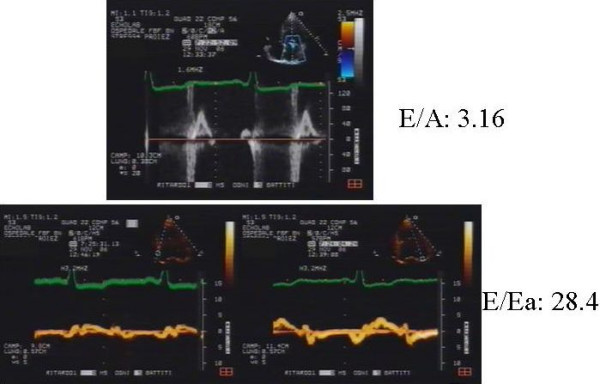
Transmitral LV filling pattern (up); mitral annulus pulsed Tissue Doppler velocity at septum (left panel) septal (down left) and lateral wall (down right) corner.

In HF patient it is very important analyzed all of the echocardiographic parameters that we can have. We considered 2 patients with chronic HF and dilated cardiomyopathy after myocardial infarction. The 2 patients had a similar ejection fraction (patient 1: 24%, patient 2: 22%) and wall motion score index (patient 1: 2.43 patient 2: 2.54). When we assessed the diastolic function, we found an abnormal relaxation LV filling pattern (Grade I) in patient 1 with a normal LV filling pressure (E/E' 9.4) (Figure 5), while patient 2 had restrictive irreversible LV filling pattern (Grade IV) and elevated LV filling pressure (E/E' 28.4), moderate mitral regurgitation and 32 ultrasound lung comets (Figure 6). During high dose dobutamine stress echo patient 1 showed a significant increase in wall motion score index (from 2.43 at baseline to 1,5 at peak stress) and in ejection fraction (from 24% at baseline to 40% at peak stress), while wall motion score index (2.54 at baseline and at peak stress) and ejection fraction (from 22% at baseline to 23% at peak stress) did not change significantly in patient 2.

Hence this approach identifies a low risk patient (patient 1) in NYHA functional class II, with normal exercise tolerance (peak oxygen consumption 15,1 ml/kg/min) and light increase in BNP levels (280 pg/ml); on the other hand, patient 2 is a high risk, in NYHA functional class III, with abnormal exercise tolerance (peak oxygen consumption 10,2 ml/kg/min) and elevated in BNP levels (1190 pg/ml).

Both of patient underwent to cardiac resynchronization therapy. At 1-year follow-up, patient 1 was a responder to resynchronization therapy: in fact it showed an improvement in symptoms (NYHA functional class I) with a significant reduction in end-systolic volume (from 137 ml to 107 ml, Δ -22%) and increase in ejection fraction (from 24% to 33%, Δ +37%). Patient 1 was a non-responder: the symptoms did not change (NYHA functional class III), without a significant reduction in end-systolic volume (from 115 ml to 102 ml, Δ -11%) and increase in ejection fraction (from 22% to 25%, Δ +13%).

## Conclusion

**Figure 7:**
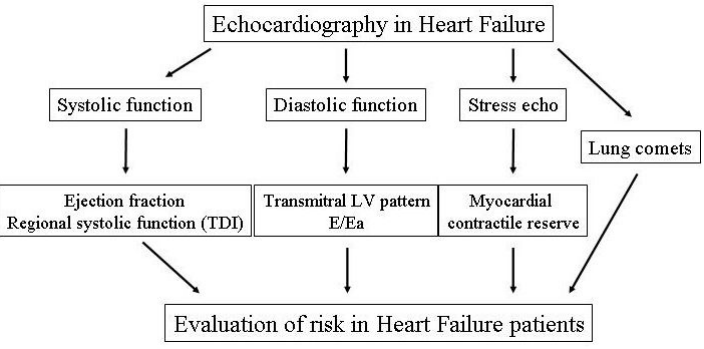
Role of echocardiography in the evaluation of risk in heart failure patients.

In HF patients, the evaluation of systolic, diastolic function and myocardial contractile reserve play a fundamental role in the risk stratification (Figure 7). In fact, as we demonstrated in the example, the evaluation of systolic function (i.e. ejection fraction), that often comes demanded like a single parameter of appraisal of the severity of HF patient, it is a part in the assessment of the risk: the highest risk is present in HF patients with heart weak, big, noisy, stiff and wet.

## References

[B1] Braunwald E (2005). Heart disease. A textbook of cardiovascular medicine.

[B2] Redfield MM (2002). Heart failure – an epidemic of uncertain proportions. N Engl J Med.

[B3] Remme WJ, McMurray JJ, Rauch B, Zannad F, Keukelaar K, Cohen-Solal A, Lopez-Sendon J, Hobbs FD, Grobbee DE, Boccanelli A, Cline C, Macarie C, Dietz R, Ruzyllo W (2005). Public awareness of heart failure in Europe: first results from SHAPE. Eur Heart J.

[B4] American Heart Association (2005). Heart Disease and Stroke Statistics: 2005 Update.

[B5] Hunt SA, Abraham WT, Chin MH, Feldman AM, Francis GS, Ganiats TG, Jessup M, Konstam MA, Mancini DM, Michl K, Oates JA, Rahko PS, Silver MA, Stevenson LW, Yancy CW (2005). ACC/AHA 2005 guideline update for the diagnosis and management of chronic heart failure in the adult: a report of the American College of Cardiology/American Heart Association Task Force on Practice Guidelines (Writing Committee to Update the 2001 Guidelines for the Evaluation and Management of Heart Failure). J Am Coll Cardiol.

[B6] Goldberg LR, Jessup M (2006). Stage B heart failure: management of asymptomatic left ventricular systolic dysfunction. Circulation.

[B7] Swedberg K, Cleland J, Dargie H, Drexler H, Follath F, Komajda M, Tavazzi L, Smiseth OA, Gavazzi A, Haverich A, Hoes A, Jaarsma T, Korewicki J, Levy S, Linde C, Lopez-Sendon JL, Nieminen MS, Pierard L, Remme WJ, Task Froce for the Diagnosis and Treatment of Chronic Heart Failure of the European Society of Cardiology (2005). Guidelines for the diagnosis and treatment of chronic heart failure: executive summary (update 2005). Eur Heart J.

[B8] Oh JK (2007). Echocardiography in heart failure: beyond diagnosis. Eur J Echocardiogr.

[B9] Picano E (2004). Sustainability of medical imaging. BMJ.

[B10] Picano E (2005). Economic and biological costs of cardiac imaging. Cardiovasc Ultrasound.

[B11] European Commission on Radiation protection 118:Referral guidelines for imaging. http://ec.europa.eu/energy/nuclear/radioprotection/publication/doc/118_en.pdf.

[B12] International Commission on Radiation Protection (2001). Radiation and your patient: a guide for medical practitioners. A web module produced by Committee 3 of the International Commission on Radiological Protection.

[B13] International Commission on Radiological Protection (1991). Radiological protection in Biomedical Research.

[B14] St John Sutton M, Pfeffer MA, Plappert T, Rouleau JL, Moyé LA, Dagenais GR, Lamas GA, Klein M, Sussex B, Goldman S (1994). Quantitative two-dimensional echocardiographic measurements are major predictors of adverse cardiovascular events after acute myocardial infarction. The protective effects of Captopril. Circulation.

[B15] Lang RM, Bierig M, Devereux RB, Flachskampf FA, Foster E, Patricia A, Pellikka PA, Picard MH, Roman MJ, Seward J, Shanewise J, Solomon S, Spencer KT, Sutton MSJ, Stewart W (2006). Recommendations for chamber quantification. Eur J Echocardiogr.

[B16] Jensen-Urstad K, Bouvier F, Hojer J, Ruiz H, Hulting J, Samad B, Thorstrand C, Jensen-Urstad M (1998). Comparison of different echocardiographic methods with radionuclide imaging for measuring LVEF during acute myocardial infarction treated by thrombolytic therapy. Am J Cardiol.

[B17] Gottdiener JS, Bednarz J, Devereux R, Gardin J, Klein A, Manning WJ, Morehead A, Kitzman D, Oh J, Quinones M, Schiller NB, Stein JH, Weissman NJ, American Society of Echocardiography (2004). American Society of Echocardiography recommendations for use of echocardiography in clinical trials. J Am Soc Echocardiogr.

[B18] King DL, Harrison MR, King DL, Gopal AS, Martin RP, DeMaria AN (1992). Improved reproducibility of left atrial and left ventricular measurements by guided three-dimensional echocardiography. J Am Coll Cardiol.

[B19] Nosir YF, Fioretti PM, Vletter WB, Boersma E, Salustri A, Postma JT, Reijs AE, Ten Cate FJ, Roelandt JR (1996). Accurate measurement of left ventricular ejection fraction by three-dimensional echocardiography. A comparison with radionuclide angiography. Circulation.

[B20] Mor-Avi V, Sugeng L, Weinert L, MacEneaney P, Caiani EG, Koch R, Salgo IS, Lang RM (2004). Fast measurement of left ventricular mass with real-time three-dimensional echocardiography: comparison with magnetic resonance imaging. Circulation.

[B21] Cerqueira MD, Weissman NJ, Dilsizian V, Jacobs AK, Kaul S, Laskey WK, Pennell DJ, Rumberger JA, Ryan T, Verani MS, American Heart Association Writing Group on Myocardial Segmentation and Registration for Cardiac Imaging (2002). Standardized myocardial segmentation and nomenclature for tomographic imaging of the heart: a statement for healthcare professionals from the Cardiac Imaging Committee of the Council on Clinical cardiology of the American Heart Association. Circulation.

[B22] Mann DL (1999). Mechanisms and models in heart failure: A combinatorial approach.. Circulation.

[B23] Grossmann W, Jones D, McLaurin LP (1975). Wall stress and patterns of hypertrophy in the human left ventricle. J Clin Invest.

[B24] Hein S, Arnon E, Kostin S, Schönburg M, Elsässer A, Polyakova V, Bauer EP, Klövekorn WP, Schaper J (2003). Progression from compensated hypertrophy to failure in the pressure-overloaded human heart structural deterioration and compensatory mechanisms. Circulation.

[B25] Grayburn PA, Appleton CP, DeMaria AN, Greenberg B, Lowes B, Oh J, Plehn JF, Rahko P, St John Sutton M, Eichhorn EJ, BEST Trial Echocardiographic Substudy Investigators (2005). Echocardiographic predictors of morbidity and mortality in patients with advanced heart failure. The Beta-blocker Evaluation of Survival Trial (BEST). J Am Coll Cardiol.

[B26] Kannel WB, Sorlie P, McNamara PM (1979). Prognosis after initial myocardial infarction: the Framingham study. Am J Cardiol.

[B27] Yiu S, Sarano M, Tribouilloy C, Seward J, Tajik A (2000). Determinants of the degree of functional mitral regurgitation in patients with systolic left ventricular dysfunction. A quantitative clinical study. Circulation.

[B28] Lamas GA, Mitchell GF, Flaker GC, Smith SC, Gersh BJ, Basta L, Moye L, Braunwald E, Pfeffer MA (1997). Clinical significance of mitral regurgitation after acute myocardial infarction. Circulation.

[B29] Lancellotti P, Gerard PL, Pierard LA (2005). Long-term outcome of patients with heart failure and dynamic functional mitral regurgitation. Eur Heart J.

[B30] Trichon BH, Felker GM, Shaw LK, Cabell CH, O'Connor CM (2003). Relation of frequency and severity of mitral regurgitation to survival among patients with left ventricular systolic dysfunction and heart failure. Am J Cardiol.

[B31] Boltwood CM, Tei C, Wong M, Shah PM (1983). Quantitative echocardiography of the mitral complex in dilated cardiomyopathy: the mechanism of functional mitral regurgitation.. Circulation.

[B32] Kwan J, Shiota T, Agler DA, Popovic ZB, Qin JX, Gillinov MA, Stewart WJ, Cosgrove DM, McCarthy PM, Thomas JD (2003). Geometric differences of the mitral apparatus between ischemic and dilated cardiomyopathy with significant mitral regurgitation. Real-time three-dimensional echocardiography study. Circulation.

[B33] Heinle SK, Tice FD, Kisslo J (1995). Effect of dobutamine stress echocardiography on mitral regurgitation. J Am Coll Cardiol.

[B34] Tunick PA, Freedberg RS, Gargiulo A, Kronzon I (1992). Exercise Doppler echocardiography as an aid to clinical decision making in mitral valve disease. J Am Soc Echocardiogr.

[B35] Lancellotti P, Lebrun F, Pierard LA (2003). Determinants of exercise-induced changes in mitral regurgitation in patients with coronary artery disease and left ventricular dysfunction. J Am Coll Cardiol.

[B36] Keren G, Laniado S, Sonnenblick EH, Lejemtel TH (1989). Dynamics of functional mitral regurgitation during dobutamine therapy in patients with severe congestive heart failure: a Doppler echocardiographic study. Am Heart J.

[B37] Lancellotti P, Troisfontaines P, Toussaint AC, Pierard LA (2003). Prognostic importance of exercise-induced changes in mitral regurgitation in patients with chronic ischemic left ventricular dysfunction. Circulation.

[B38] European Study group on Diastolic Heart Failure (1998). How to diagnose diastolic heart failure. European Study Group on Diastolic Heart Failure.. Eur Heart J.

[B39] Appleton CP, Hatle LK, Popp RL (1988). Relation of transmitral flow velocity patterns of left ventricular diastolic function: new insights from a combined hemodynamic and Doppler echocardiographic study. J Am Coll Cardiol.

[B40] Cohen GI, Pietrolungo JF, Thomas JD, Klein AL (1996). A practical guide to assessment of ventricular diastolic function using Doppler echocardiography. J Am Coll Cardiol.

[B41] Garcia MJ, Thomas JD, Klein AL (1998). New Doppler echocardiographic application for the study of diastolic function. J Am Coll Cardiol.

[B42] Nishimura RA, Appleton CP, Redfield MM, Ilstrup DM, Holmes DR, Tajik AJ (1996). Noninvasive Doppler echocardiographic evaluation of left ventricular filling pressure in patients with cardiomyophaties: a simultaneous Doppler echocardiographic and cardiac catheterization study. J Am Coll Cardiol.

[B43] Nishimura RA, Tajik AJ (1997). Evaluation of diastolic filling of left ventricle in health and disease: Doppler echocardiography is the clinician's Rosetta Stone.. J Am Coll Cardiol.

[B44] Hansen A, Haass M, Zugck C, Krueger C, Unnebrink K, Zimmermann R, Kuebler W, Kuecherer H (2001). Prognostic value of Doppler echocardiographic mitral inflow patterns: implications for risk stratification in patients with congestive heart failure. J Am Coll Cardiol.

[B45] Giannuzzi P, Temporelli PL, Bosimini E, Silva P, Imparato A, Corra U, Galli M, Giordano A (1996). Independent and incremental prognostic value of Doppler-derived mitral deceleration time of early filling in both symptomatic and asymptomatic patients with left ventricular dysfunction. J Am Coll Cardiol.

[B46] Pinamonti B, Di Lenarda A, Sinagra G, Camerini F (1993). Restrictive left ventricular filling pattern in dilated cardiomyopathy assessed by Doppler echocardiography: clinical, echocardiographic and hemodynamic correlations and prognostic implications. J Am Coll Cardiol.

[B47] Temporelli PL, Corra U, Imparato A, Bosimini E, Scapellato F, Giannuzzi P (1998). Reversible restrictive left ventricular diastolic filling with optimized oral therapy predicts a more favorable prognosis in patients with chronic heart failure. J Am Coll Cardiol.

[B48] Yu CMMD, MD, Sanderson JE, Marwick TH, Oh JK (2007). Tissue Doppler Imaging. A New Prognosticator for Cardiovascular Diseases. J Am Coll Cardiol.

[B49] Nagueh S, Sun H, Kopelen H, Middleton K, Khoury D (2001). Hemodynamic determinants of the mitral annulus diastolic velocities by tissue Doppler. J Am Coll Cardiol.

[B50] Yu CM, Lin H, Yang H, Shun-Ling Kong SL, Zhang Q, Wai-Luen Lee S (2002). Progression of systolic abnormalities in patients with isolated" diastolic heart failure and diastolic dysfunction. Circulation.

[B51] Nagueh SF, Middleton KJ, Kopelen HA, Zoghbi WA, Quinones MA (1997). Doppler tissue imaging: a noninvasive technique for evaluation of left ventricular relaxation and estimation of filling pressures. J Am Coll Cardiol.

[B52] Halperin BD, Feeley TW, Mihm FG, Chiles C, Guthaner DF, Blank NE (1985). Evaluation of the portable chest roentgenogram for quantitating extravascular lung water in critically ill adults. Chest.

[B53] Eisenberg PR, Hansbrough JR, Anderson D, Schuster DP (1987). A prospective study of lung water measurements during patient management in an intensive care unit. Am Rev Respir Dis.

[B54] Targhetta R, Chavagneux R, Bourgeois JM, Dauzat M, Balmes P, Pourcelot L (1992). Sonographic approach to diagnosing pulmonary consolidation. J Ultrasound Med.

[B55] Ziskin MC, Thickman DI, Goldenberg NJ, Lapayowker MS, Becker JM (1982). The comet tail artifact. J Ultrasound Med.

[B56] Picano E, Frassi F, Agricola E, Gligorova S, Gargani L, Mottola G (2006). Ultrasound lung comets: a clinically useful sign of extravascular lung water. J Am Soc Echocardiogr.

[B57] Jambrik Z, Monti S, Coppola V, Agricola E, Mottola G, Miniati M, Picano E (2004). Usefulness of ultrasound lung comets as a nonradiologic sign of extravascular lung water. Am J Cardiol.

[B58] Agricola E, Bove T, Oppizzi M, Marino G, Zangrillo A, Marginato A, Picano E (2005). "Ultrasound comet-tail images": a marker of pulmonary edema. A comparative study with wedge pressure and extravascular lung water. Chest.

[B59] Frassi F, Gargani L, Gligorova S, Ciampi Q, Mottola G, Picano E (2006). Clinical and echocardiographic determinants of ultrasound lung comets. Eur J Echocardiography.

[B60] Frassi F, Gargani L, Tesorio P, Raciti M, Mottola G, Picano E (2007). Prognostic value of extravascular lung water assessed with ultrasound lung comets by chest sonography in patients with dyspnea and/or chest pain. J Cardiac Fail.

[B61] Picano E (1992). Stress echocardiography. From pathophysiological toy to diagnostic tool. Circulation.

[B62] Pratali L, Picano E, Otaševiæ P, Vigna C, Palinkas A, Cortigiani L, Dodi C, Bojiæ D, Varga A, Csanady M, Landi P (2001). Prognostic significance of the dobutamine echocardiography test in idiopathic dilated cardiomyopathy. Am J Cardiol.

[B63] Mannor A, Shneeweiss A (1997). Prognostic value of noninvasively obtained left ventricular contractile reserve in patients with severe heart failure. J Am Coll Cardiol.

[B64] Agricola E, Oppizzi M, Pisani M, Margonato A (2004). Stress echocardiography in heart failure. Cardiovasc Ultrasound.

[B65] Drozdz J, Krzeminska-Pakula M, Plewka M, Ciesielczyk M, Kasprzak JD (2002). Prognostic value of low-dose dobutamine echocardiography in patients with idiopathic dilated cardiomyopathy. Chest.

[B66] Borow KM, Lang RM, Neumann A, Carrol JD, Rajfer SI (1988). Physiologic mechanisms governing hemodynamic responses to positive inotropic therapy in patients with dilated cardiomyopathy. Circulation.

[B67] Grossman W, Baim DS, Grossman W (1996). Evaluation of systolic and diastolic function of the myocardium. cardiac catheterization, angiography and intervention.

[B68] Viquerat CE, Daly P, Swedberg K (1985). Endogenous cateholamine levels in chronic heart failure: relation to the severity of hemodynamic abnormalities. Am J Med.

[B69] Carrol JD, Lang RM, Neumann A, Borow KM, Rajfer SI (1986). The differential effects of positive inotropic and vasodilator therapy in patients with congestive cardiomyopathy. Circulation.

[B70] Otasević P, Popović ZB, Vasiljević JD, Vidaković R, Pratali L, Vlahović A, Nesković AN (2005). Relation of myocardial histomorphometric features and left ventricular contractile reserve assessed by high-dose dobutamine stress echocardiography in patients with idiopathic dilated cardiomyopathy. Eur J Heart Fail.

[B71] Mulieri LA, Hasenfuss G, Leavitt B, Allen PD, Alpert NR (1992). Altered myocardial force-frequency relation in human heart failure. Circulation.

[B72] Lewartowski B, Pytkowski B (1987). Cellular mechanism of the relationship between myocardial force and frequency of contractions. Prog Biophys Mol Biol.

[B73] Bombardini T, Correia MJ, Cicerone C, Agricola E, Ripoli A, Picano E (2003). Force-frequency relationship in the echocardiography laboratory: a noninvasive assessment of Bowditch treppe?. J Am Soc Echocardiogr.

[B74] Bombardini T, Agrusta M, Natsvlishvili N, Solimene F, Pap R, Coltorti F, Varga A, Mottola G, Picano E (2005). Noninvasive assessment of left ventricular contractility by pacemaker stress echocardiography. Eur J Heart Failure.

[B75] Bombardini T (2005). Myocardial contractility in the echo lab: molecular, cellular and pathophysiological basis. Cardiovasc Ultrasound.

[B76] Inagaki M, Yokota M, Izawa H, Ishiki R, Nagata K, Iwase M, Yamada Y, Koide M, Sobue T (1999). Impaired force-frequency relations in patients with hypertensive left ventricular hypertrophy. Circulation.

[B77] Picano E, Sicari R, Landi P, Cortigiani L, Coletta C, Galati A, Heyman J, Mattioli R, Previtali M, Mathias W, Dodi C, Minardi G, Lowenstein J, Seveso G, Pingitore A, Salustri A, Raciti M (1998). Prognostic value of myocardial viability in medically treated patients with global left ventricular dysfunction early after an acute uncomplicated myocardial infarction: a dobutamine stress echocardiographic study. Circulation.

[B78] Kitaoka H, Takata J, Yabe T, Hitomi N, Furuno T, Doi YL (1999). Low dose dobutamine stress echocardiography predicts the improvement of left ventricular systolic function in dilated cardiomyopathy. Heart.

[B79] Jourdain P, Funck F, Bellorini M, Josset C, Piednoir C, Pons N, Loiret J, Guillard N, Thebault B, Desnos M (2002). Myocardial contractile reserve under low doses of dobutamine and improvement of left ventricular ejection fraction with treatment by carvedilol. Eur J Heart Fail.

[B80] Cigarroa CG, deFilippi CR, Brickner ME, Alvarez LG, Wait MA, Grayburn PA (1993). Dobutamine stress echocardiography identifies hibernating myocardium and predicts recovery of left ventricular function after coronary revascularization. Circulation.

[B81] Da Costa A, Thevenin J, Roche F, Faure E, Romeyer-Bouchard C, Messier M, Convert G, Barthelemy JC, Isaaz K (2006). Prospective validation of stress echocardiography as an identifier of cardiac resynchronization therapy responders. Heart Rhythm.

